# Disability, physical activity, and health-related quality of life in Australian adults: An investigation using 19 waves of a longitudinal cohort

**DOI:** 10.1371/journal.pone.0268304

**Published:** 2022-05-12

**Authors:** Syed Afroz Keramat, Benojir Ahammed, Aliu Mohammed, Abdul-Aziz Seidu, Fariha Farjana, Rubayyat Hashmi, Kabir Ahmad, Rezwanul Haque, Sazia Ahmed, Mohammad Afshar Ali, Bright Opoku Ahinkorah

**Affiliations:** 1 Economics Discipline, Social Science School, Khulna University, Khulna, Bangladesh; 2 School of Business, University of Southern Queensland, Toowoomba, QLD, Australia; 3 Centre for Health Services Research, University of Queensland, Brisbane Australia; 4 Statistics Discipline, Science, Engineering, Technology School, Khulna University, Khulna, Bangladesh; 5 Department of Health, Physical Education, and Recreation, University of Cape Coast, Cape Coast, Ghana; 6 Centre For Gender and Advocacy, Takoradi Technical University, Takoradi, Ghana; 7 College of Public Health, Medical and Veterinary Sciences, James Cook University, Townsville Australia; 8 QUT Business School, Queensland University of Technology, Brisbane, QLD, Australia; 9 Quality Use of Medicines and Pharmacy Research Centre (QUMPRC), Clinical and Health Sciences, University of South Australia, Adelaide, South Australia; 10 Department of Economics, Jagannath University, Dhaka, Bangladesh; 11 School of Public Health, University of Technology Sydney, Sydney, Australia; University of Alberta, CANADA

## Abstract

**Background:**

Any form of long-term physical or mental impairment might negatively influence health-related quality of life (HRQoL). HRQoL, as an independent concept, covers a wide range of characteristics that includes physical, mental, social, and spiritual functions. People with disabilities are continuously exposed to multiple barriers that deteriorate their HRQoL. It also creates impairment in performing physical activities. However, experts opine regular physical exercise as an intervention to help disabled people. This research aims to investigate the association between disability and physical activity with HRQoL among the adult population in Australia.

**Design:**

A retrospective cohort study.

**Methods:**

This study utilized the most recent 19 waves of data (2002–2020) from the nationally representative Household, Income and Labour Dynamics in Australia (HILDA) survey. Component summary scores such as physical component summary (PCS) and mental component summary (MCS), and SF-6D utility scores were utilized to measure HRQoL. Random-effects GLS regression technique was fitted to estimate the association between disability and physical activity with HRQoL, after adjusting for a range of socio-demographic and health-related characteristics.

**Results:**

Disability was negatively associated with the PCS (-5.95), MCS (-2.70) and SF-6D (-0.060) compared with non-disabled counterparts. However, respondents engaged in the recommended level of physical activity had substantial gain in PCS (b = 0.96), MCS (1.57), and SF-6D (0.021) scores. Besides, the results showed that performing the recommended level of physical activity in the presence of disability has lessen the negative effect of disability/ positive moderating effect of physical activity on PCS, MCS, and SF-6D scores by 1.84 points, 0.82 points, and 0.013 percentage points, respectively.

**Conclusion:**

This study found an inverse association between disability and HRQoL among Australian adults. However, physical activity was associated with improved HRQoL. Therefore, public health interventions, such as the orientation of physical activities, have a higher potential to dwindle the burden regarding HRQoL.

## Introduction

Globally, disability has evolved as a major public health concern. The number of people living with a disability is increasing dramatically. Population ageing and the increase in chronic diseases are dominant factors behind inflated disabilities in the past few decades [1]. According to the World Health Organization (WHO), more than one billion people worldwide live with some form of disability, and about 190 million adults living with disability have considerable functional difficulties [2]. In Australia, an estimated 4.4 million people (18% of Australians) are living with disability, and of which nearly 1.4 million (32%) are living with a severe or profound form of disability [3]. Besides, a recent study discovered a 28 percent prevalence of self-reported disability among Australian adults [4]. Available evidence suggests that people living with disabilities have an increased risk for chronic diseases and poor health-related quality of life (HRQoL), mainly attributable to a high level of sedentary behaviour among people with disabilities [5, 6]. Additionally, evidence suggests that persons with disabilities are more likely to rate their physical and mental health as poor or fair [7]. Apart from the direct costs, disability has indirect costs in the form of increased absenteeism [8], rising presenteeism [9], low job satisfaction [10], and high workplace discrimination [11].

Disability is a multidimensional concept denoting impairment in body functioning or structure, limitations in activity performance, and restrictions in participation [1]. Generally, people with disabilities have poor HRQoL [12], which is described as an individual’s perceived level of physical, mental and social functioning [13]. Existing evidence suggests that people with disabilities are highly susceptible to chronic diseases, including cardiovascular diseases [14] and mental disorders [15]. The high prevalence of chronic diseases and comorbidities among persons with a disability has been attributed to increased levels of sedentary behaviour or physical inactivity [16, 17]. In Australia, for instance, sedentary behaviour is the second leading contributor to the burden of cancer [18], which disproportionately affects persons with disabilities [19]. It is suggested that physical, psychological, and social functioning limitations among persons with disabilities promote sedentary behaviour and minimize physical activity [20].

Regular physical activity is associated with significant improvement in physical and mental health. Existing research indicates that engaging in the recommended level of physical activity helps prevent disability [4]. It also reduces the risk for chronic diseases and comorbidities, especially among people with disability [15, 21]. For instance, a meta-analysis of 18 randomized controlled trials reported that physical activity significantly improved the cognitive function and quality of life of people suffering from dementia [22]. Likewise, a meta-analysis of 39 randomized controlled trials of adults with mental illnesses found that physical activity significantly reduced depressive symptoms among people with mental illnesses [23]. A randomized controlled trial of persons with spinal cord injury found that regular exercise improved muscular strength, reduced pain and depression, and improved quality of life among the experimental group [24]. Another recent study reported that substituting 1 hour per day of sedentary behaviour with physical activity significantly improved the physical functioning of colorectal cancer survivors [25].

Although physical activity is vital to the health and quality of life of persons with disabilities, most physical activity programs do not target people with disabilities [14]. Many people with disabilities continue to experience multiple barriers to participate in physical activities [21]. Meanwhile, compared to the general population, people with disabilities tend to gain enormously from physical activities, especially in improving cognition and physical functioning [22, 24]. Therefore, the WHO included physical activity recommendations for people with disabilities in its latest guidelines on physical activity and sedentary behaviour [21]. Despite several efforts to increase physical activity among Australians, the rate remains low, especially among people with disabilities [3]. For instance, a recent survey revealed that 72% of adults with disabilities in Australia do not perform the recommended level of physical activity [3].

Previous studies primarily targeted the aged population [26] or individuals with disabilities associated with specific conditions such as multiple sclerosis [27] or spinal cord injury [28] while capturing the relationships between disability and HRQoL. Thus, there is a limited information on the longitudinal association of disability and physical activity with HRQoL. Therefore, the present study examines the association between disability and physical activity with HRQoL among the Australian adult population using longitudinal data from the Household, Income and Labour Dynamics in Australia (HILDA) survey. Understanding how physical activity and disability are related to HRQoL could help form policies to improve the HRQoL and may serve as the input for future economic evaluation for decision-making while allocating resources for effective health interventions.

## Methods

### Data description and sample

The study used data from the HILDA survey which commenced in 2001. The dataset covers information on subjects’ wealth, labour market outcomes, household and family relationships, fertility, health, and education. A multistage sampling approach was used to select an initial sample. Firstly, a probability proportional to size sampling technique was initiated to select 488 Census Collection Districts (CDs). Each of the districts covers approximately 200–250 households. Secondly, a sample of 22–34 dwellings was randomly selected from each of the CDs. Finally, a maximum of three households from each dwelling was selected, resulting in a total of 12,252 households. Since 2001, the annual data accumulation covers household members aged 15 years and over as a sample. Face-to-face interviews and telephone interviews by trained enumerators were used for data collection. A self-completed questionnaire following the University of Melbourne’s ethical guidelines is used in this regard. The sample was further expanded over time. It includes any child born or adopted by groups of respondents or by any new household member resulting from adjustments of the composition of the originating households. Therefore, the annual coverage of the survey is more than 17,000 Australian adults. The details of the sampling procedure, study design and data collection strategies of the waves have been explained elsewhere [29].

**Fig 1:**
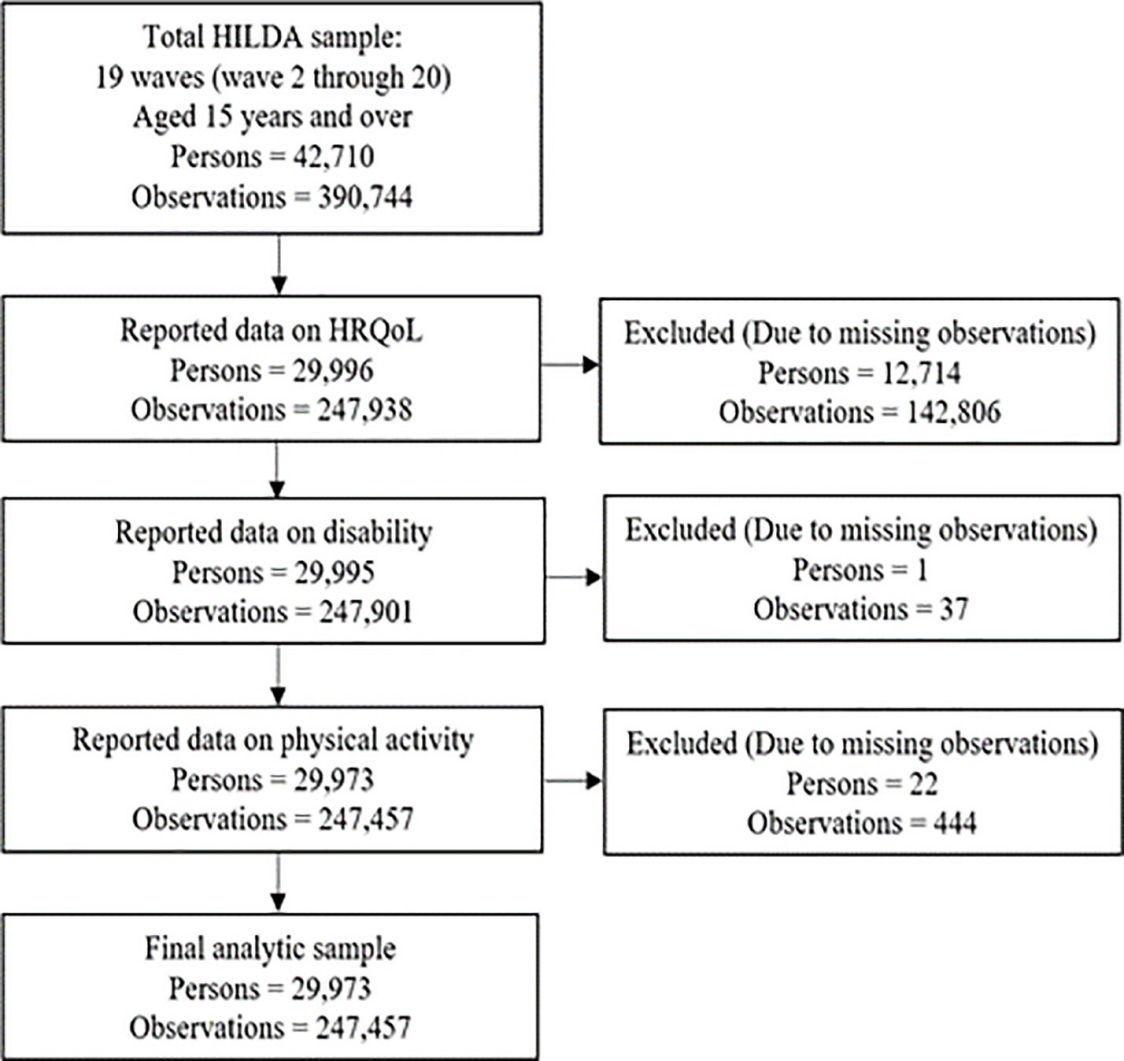
Participants flow into the analytic sample, and missing data.

Fig 1 shows participants’ flow into the final analytic sample and missing data. This study utilized 19 waves of data (waves 2 through 20), spanning 2002 to 2020. These 19 waves contain detailed information on participants’ socio-demographic, disability status, and physical activity, along with HRQoL information collected through the SF-36 questionnaire. To avoid the potential bias, missing observations on the outcome variable (HRQoL) and main variables of interest (disability and physical activity) were excluded. The final analytic sample consists of 247,457 person-year observations from 29,973 unique participants.

### Outcome variable

The health-related quality of life (HRQoL) was the outcome variable of this study. Information on HRQoL was collected through the SF-36 questionnaire. It contains 36 questions. The taxonomy of SF-36 encompasses three levels, e.g., items, aggregate scales, and summary measures. The items are mutually exclusive. One single item is considered for scoring one scale only. These items help with the formation of eight aggregate scales, i.e. physical functioning (PF), role physical (RP), role emotional (RE), social functioning (SF), mental health (MH), vitality (VT), bodily pain (BP), and general health (GH) [30]. There is no avenue for trading off these eight domains. Each of them has unique importance. Basically, items are standardized by applying a scoring algorithm that ranges between 0 to 100. A higher score indicates better HRQoL [31]. In the last taxonomy, these eight dimensions of HRQoL are transformed into two higher-ordered clusters of summary measure, i.e., the physical component summary (PCS) and the mental component summary (MCS).

The PCS and MCS transformation from the eight aggregate domains are ensured by deploying an orthogonal factor model. Here, the physical domains were weighted positively, and the mental domains were weighted negatively (vice versa). It implies a better state of mental health lowers the PCS and vice-versa [13]. Hence, a well recommended standard scoring algorithm was followed and standardized by linear Z-score transformation with a mean of 50 ± 10 (SD). The value of PCS and MCS ranged between 4.54 to 76.09, and -1.21 to 76.19, respectively. A higher score indicates better HRQoL [32, 33].

This study also considers SF-6D, an alternative preference-based generic measure of health utility index. It estimates the respondent’s choice for health status. It enables the estimation of a utility score by using SF-36 responses [34]. This is an ‘off-the-shelf’ measure retrieved from a set of readily available preference values from general people. ‘Standard Gamble’ (SG) technique is applied for the preference elicitation. This method measures the health utility from six different dimensions (constructed from SF-36), namely, physical functioning, physical role limitations, emotional role limitations, impairment of social functioning, body pain, and vitality [35]. These six domains conceive 11 items retrieved from SF-36. SF-6D considers the willingness of an individual to accept the risk of death to current living discomfort (or enjoy living in good health status with no physical and mental impairment). The SF-6D score ranges from 0.29 to 1. Here, 1 is considered as full health (all the dimensions are in the best state), and 0.29 demonstrates the worst health condition (equivalent to death) [36].

### Exposure variables

Disability and physical activity are the primary exposures. HILDA survey defines disability by following the guidelines of the International Classification of Functioning, Disability and Health (ICF) under the WHO framework [8, 37]. A show card listing 17 types of disability was used for assessing the presence of disability [8]. The HILDA survey collects information on participants’ disability status by asking, “is there any form of physical impairment hindering your everyday activities that lasted or expected to last for six months or more?” [38]. The responses were taken in binary form (yes versus no).

Another key variable of interest is physical activity. Each of the waves of the HILDA survey to date includes data on physical activity. It intends to assess the respondent’s extent of engagement in physical activity [39]. Each year a self-completion questionnaire is formed to calculate the frequency of physical activity/ per week. Participants were asked, ‘‘In general, how often do you participate in moderate or intensive physical activity for at least 30 minutes?” [33]. The prospective answers were pre-coded into six categories: ‘no involvement at all; less than once a week; 1 or 2 times a week; three times a week; more than thrice a week (but not every day) and every day [40]. Such clustering targets to capture the optimal frequency of Australian adults’ involvement in physical activities. The categories were further collapsed into two clusters: less than the recommended level (no involvement at all, less than once, 1–2 times, and three times a week), and recommended level (more than three times a week, and every day).

### Potential confounding factors

This study included potential confounding factors following previous literature on HRQoL, disability, and physical activity [41, 42]. The potential confounding factors plugged in the statistical analyses were age, gender, relationship status, highest education level completed, household yearly disposable income, labour force status, Indigenous status, region of residence, smoking habit, and alcohol consumption.

Table 1 presents the set of potential confounding factors with their nature and categories. For example, age was initially a continuous variable but collapsed into four categories: 15–29, 30–44, 45–59, and ≥ 60 years. Other confounding factors considered for the present study were gender (male versus female), relationship status (single versus couple), education (year 12 and below, certificate courses, and university degrees), household income (1^st^ to 5^th^ quintile, which indicates lower income group to upper-income group in order), labour force status (employed, versus unemployed or not in the labour force), Indigenous status (not of Indigenous origin versus Indigenous origin), and region of residence (major city versus regional or remote area). Further, the research considered the inclusion of two health-related characteristics, i.e., smoking habit (never smoked, former smoker, and current smoker), and alcohol consumption (never drunk, ex-drinker, and current drinker).

**Table 1:** Description of the control variables.

Variables	Description
Age	15–29, 30–44, 45–59, and ≥ 60 years.
Gender	Male and female.
Relationship status	Single (never married and not living with someone in a relationship, separated but not divorced, divorced, and widowed), and couple (married in a registered marriage, and never married but living with someone in a relationship).
Highest education level completed	Year 12 and below (year 12, and year 11 and below), certificate courses (advance diploma or diploma, and certificate III or IV), and university degrees (postgraduate—masters or doctorate, graduate diploma or certificate, bachelor or honours).
Household yearly disposable income	Quintile 1 (poorest), quintile 2 (poorer), quintile 3 (middle), quintile 4 (richer), and quintile 5 (richest).
Labor force status	Employed, and unemployed or not in the labour force (NLF).
Indigenous status	Not of Indigenous origin, and Indigenous origin (Aboriginal, Torres Strait Islander, and both Aboriginal and Torres Strait Islander).
Region of residence	Major city, and regional or remote area (inner regional, outer regional, remote and very remote Australia).
Smoking status	Never smoked, former smoker and current smoker (smoke daily, smoke at least weekly, and smoke less often than weekly).
Alcohol consumption	Never drunk, ex-drinker, current drinker (only rarely, 1–2 days, 2–3 days, 3–4 days, 5–6 days per week and every day).

### Estimation strategy

A longitudinal data covering 19 waves consisting of 29,973 (247,457 person-year observations) Australian de-identified adults were studied in this analysis. This research reported frequency (n), mean and standard deviation (SD) for the continuous variable, and percentage (%) for the categorical variables. The summary measures (PCS and MCS) and SF-6D utility score, as well as the scores of the eight dimensions of SF-36, were explored from the disability and physical activity lens. This study formed the following multivariate regression model to investigate the association between disability and physical activity with HRQoL.


$${HRQoL}_{it}=\alpha_{i}+{\beta_{1}Disability}_{it}+{\beta_{2}Physical\;activity}_{it}+{\Upsilon_{i}\Sigma X}_{it}+\mu_{it}+\varepsilon_{it}$$ (1)

In Eq 1, HRQoL_*it*_ stands for i^th^ respondents’ health-related summary measures of life quality over the t^th^ time horizon (2002 to 2020). It covers PCS, MCS, and the health utility index (SF-6D), as well as the subscales of SF-36. α_i_ (i = 1 to n) refers to the unknown intercept for each entity (n entity-specific intercepts). Disability_it_ and physical activity_it_ delineate the key variables of interests, disability status and physical activity, respectively, where i = entity, t = time, and β1 and β2 are the coefficients for that exposures. X_it_ demonstrates other confounding factors. μ_it_ refers to between-entity error and ε_it_ indicates within-entity error. This analysis fitted eleven different models, particularized by the following outcome variables: PCS, MCS, SF-6D, PF, RP, RE, SF, MH, VT, BP, and GH.

Based on the nature of the dependent variables, this study deployed the random-effects GLS regression technique. Interpretation of the coefficients obtained from the random-effects GLS regression technique are tricky since it captures both the within-entity and between-entity effects. For the present study, the regression results will show the average effect of disability and physical activity over HRQoL when disability and physical activity change across time and between persons by one unit. This study considers a variable statistically significant if the p-value is <0.05. Stata version 17.0 (Stata SE 17, College Station, TX: StataCorp LLC, USA) was used to accomplish the analysis of this study.

### Ethics approval

The HILDA survey commenced in 2001, and since then, the survey has been conducted annually following the University of Melbourne’s ethical guidelines. This research project has been approved by the Human Research Ethics Committee of The University of Melbourne. The ethics ID number of the research project (HILDA Survey) is 1647030. This paper uses unit record data from the HILDA conducted by the Australian Government Department of Social Services (DSS). This study did not require ethical approval as the analysis used only de-identified existing unit record data from the HILDA survey.

## Results

Table 2 shows the baseline, final, and pooled socio-demographic and health-related characteristics of the study sample (persons = 29,973; observations = 247,457). Over 50% of the participants were aged between 15–44 years, were female (53%), and were coupled (60%). Of the total, 25% had university qualifications, 65% were employed, 97% were non-Indigenous, 66% lived in major cities, 54% never smoked, and 82% currently drink alcohol (pooled in all waves).

**Table 2:** Distribution socio-demographic, and health-related characteristics: Baseline, final and pooled across all waves (persons = 29,973; observations = 247,457).

Characteristics	Baseline wave (2002)		Final wave(2020)		Pooled in all waves(2002 through 2020)	
	n	%	n	%	n	%
**Socio-demographic characteristics**						
**Age**						
15–29 years	2,730	24.71	3,630	24.78	64,039	25.88
30–44 years	3,543	32.06	3,737	25.51	64,062	25.89
45–59 years	2,713	24.55	3,352	22.88	61,817	24.98
≥ 60 years	2,064	18.68	3,930	26.83	57,539	23.25
**Gender**						
Male	5,258	47.58	6,720	45.87	116,069	46.90
Female	5,792	52.42	7,929	54.13	131,388	53.10
**Relationship status**						
Single	4,294	38.86	5,841	39.87	99,323	40.14
Couple	6,756	61.14	8,808	60.13	148,134	59.86
**Highest education level completed**						
Year 12 and below	5,983	54.14	5,517	37.66	111,403	45.02
Certificate courses	2,908	26.32	4,833	32.99	75,280	30.42
University degrees	2,159	19.54	4,299	29.35	60,774	24.56
**Household yearly disposable income**						
Quintile 1 (Poorest)	2,211	20	2,931	20.01	49,495	20
Quintile 2	2,211	20.01	2,929	19.99	49,488	20
Quintile 3	2,209	19.99	2,931	20.01	49,492	20
Quintile 4	2,210	20	2,930	20	49,491	20
Quintile 5 (Richest)	2,209	19.99	2,928	20	49,491	20
**Labor force status**						
Employed	7,085	64.12	9,175	62.63	159,674	64.53
Unemployed or NLF	3,965	35.88	5,474	37.37	87,783	35.47
**Indigenous status**						
Not of Indigenous origin	10,791	97.66	14,114	96.35	240,093	97.03
Indigenous origin	258	2.34	535	3.65	7,361	2.97
**Region of residence**						
Major city	7,019	63.52	9,649	65.87	162,500	65.67
**Regional or remote area**	4,031	36.48	5,000	34.13	84,957	34.33
**Health-related characteristics**						
Smoking status						
Never smoked	5,383	48.71	8,402	57.36	133,143	53.80
Ex-smoker	2,986	27.02	3,987	27.22	67,363	27.22
Current smoker	2,681	24.26	2,260	15.43	46,951	18.97
**Alcohol consumption**						
Never drunk	1,154	10.44	1,512	10.32	26,562	10.73
Ex-drinker	644	5.83	1,402	9.57	18,818	7.60
Current drinker	9,252	83.73	11,735	80.11	202,077	81.66

Table 3 displays the distribution of the analytic sample’s subjective health scores, as well as their disability and physical activity status. The mean PCS, MCS, and SF-6D scores derived from the SF-36 were 49.39, 48.35, and 0.76, respectively. The results also show that the average score on four of the eight dimensions of the SF-36 was below 76 points: MH (73.75), VT (59.41), BP (72.91), and GH (67.96). As can be seen, 27% of adults have some form of disability, and nearly 34% of the study sample performed recommended level of physical activities per week (pooled in all waves).

**Table 3:** Distribution of subjective health scores, disability status and physical activity: Baseline, final and pooled across all waves (persons = 29,973; observations = 247,457).

Variables	Baseline wave (2002)		Final wave (2020)		Pooled in all waves (2002 through 2020)	
	n	Mean (SD)	n	Mean (SD)	n	Mean (SD)
**SF-36 domain scores**						
Physical functioning	11,050	83.70 (22.65)	14,649	84.19 (22.89)	247,457	83.66 (23.15)
Role physical	11,050	79.88 (34.97)	14,649	76.67 (37.05)	247,457	78.81 (36.16)
Role emotional	11,050	83.37 (32.12)	14,649	75.58 (37.53)	247,457	82.27 (33.51)
Social functioning	11,050	82.67 (23.19)	14,649	79.99 (24.54)	247,457	82.19 (23.57)
Mental health	11,050	74.33 (17.13)	14,649	70.67 (18.43)	247,457	73.75 (17.40)
Vitality	11,050	60.82 (19.81)	14,649	56.38 (20.74)	247,457	59.41 (19.97)
Bodily pain	11,050	74.90 (24.14)	14,649	71.86 (23.52)	247,457	72.91 (23.98)
General health	11,050	69.77 (20.96)	14,649	66.42 (20.50)	247,457	67.96 (20.94)
**SF-36 component summary scores**						
PCS	11,050	49.82 (10.21)	14,649	49.81 (10.63)	247,457	49.39 (10.42)
MCS	11,050	48.80 (10.31)	14,649	46.09 (11.55)	247,457	48.35 (10.65)
SF-6D	11,050	0.76 (0.12)	14,649	0.74 (0.12)	247,457	0.76 (0.12)
**Disability (% observations)**						
No	8,762	79.29	10,341	70.59	179,973	72.73
Yes	2,288	20.71	4,308	29.41	67,484	27.27
**Physical activity** **(% observations)**						
Less than recommended level	7,258	65.68	9,573	65.35	163,850	66.21
Recommended level	3,792	34.32	5,076	34.65	83,607	33.79

**Fig 2:**
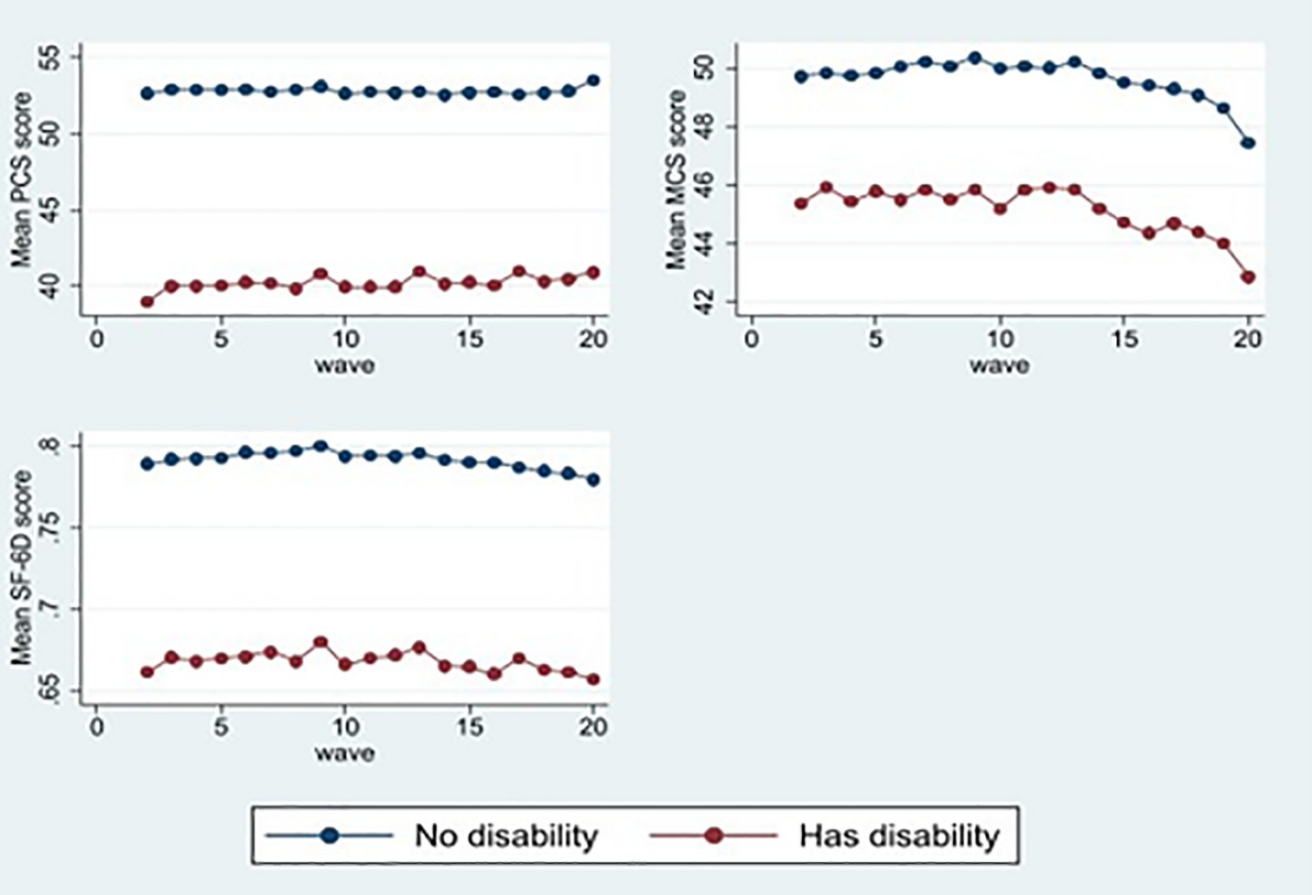
Mean SF-36 component summary scores and SF-6D utility score by disability status, waves 2002–2020.

Fig 2 displays the mean scores of the measures of HRQoL by the respondent’s disability status in each wave. Substantially low mean scores in the SF-36 subscales (8 dimensions), summary measures (PCS and MCS), and health utility index (SF-6D) have been observed among respondents who had a disability. For example, the mean PCS, MCS, and SF-6D scores among disabled participants (40.26, 45.06, and 0.67, respectively) are much lower compared to their counterparts without disability (52.82, 49.59, and 0.79, respectively) in wave 20.

**Fig 3:**
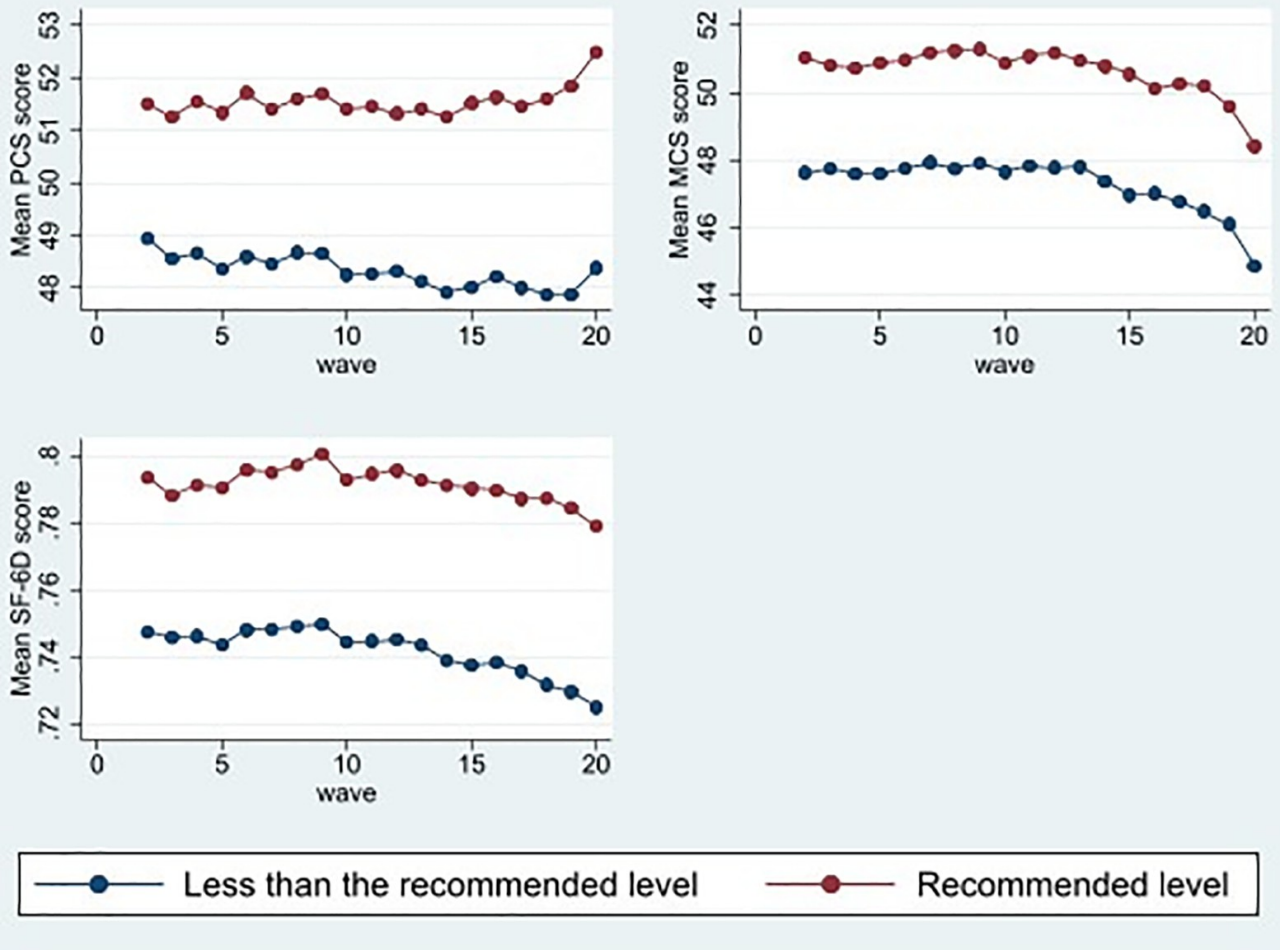
Mean SF-36 component summary scores and SF-6D utility score by physical activity, waves 2002–2020.

Fig 3 presents the mean PCS, MCS, and SF-6D utility scores by physical activity in each wave. The figure clearly illustrates that participants who undertook recommended level of physical activity had significantly higher average PCS, MCS, and SF-6D scores than those who engaged in less than the recommended level of physical activity per week. For example, the mean PCS, MCS, and SF-6D scores among the participants performing recommended level of physical activity (51.56, 50.58, and 0.79, respectively) are much higher compared to their counterparts engaged in less than the recommended level of physical activity per week (48.26, 47.21, and 0.74, respectively) in wave 20. Annual (waves 2002–2020) mean SF-36 domains score by disability status and physical activity were presented in S1 Appendix.

Table 4 represents the random-effects GLS estimation of the association between disability and physical activity with the SF-36 component summary measures and health utility index. The results revealed that PCS (b = - 5.95) and MCS (b = - 2.70) scores declined by 5.95 and 2.70 units among respondents with disability compared with non-disabled counterparts. Besides, the results showed that disabled people scored six percentage points lower on the SF-6D scale (b = -0.060) compared with peers without disability. However, the estimates demonstrate a statistically significant and positive association between undertaking the recommended level of physical activity and HRQoL. For example, performing the recommended level of physical activity per week was associated with substantial gains in PCS (b = 0.96), MCS (1.57), and SF-6D (0.021) scores. Table 4 also reports a statistically significant association between the interaction of disability and physical activity with component summary measures and health utility index. The results showed that PCS, MCS and SF-6D scores have increased in disabled adults performing the recommended level of physical activity by 1.84 points, 0.82 points, and 0.013 percentage points, respectively, than their non-disabled counterparts engaged in less than the recommended level of physical activity.

**Table 4:** The relationship between disability and physical activity with the SF-36 component summary scores and SF-6D utility score, random-effects GLS regression.

Variables	Model 1: PCS	Model 2: MCS	Model 3: SF-6D
	β (95% CI),	β (95% CI),	β (95% CI),
	P-value	P-value	P-value
**Disability**			
No (ref)			
Yes	**-5.95 (-6.04, -5.86),** **<0.001**	**-2.70 (-2.80, -2.59),** **<0.001**	**-0.060 (-0.061, -0.059),** **<0.001**
**Physical activity**			
Less than recommended level (ref)			
Recommended level	**0.96 (0.88, 1.03),** **<0.001**	**1.57 (1.49, 1.66),** **<0.001**	**0.021 (0.020, 0.022),** **<0.001**
**Interaction terms (Disability × Physical activity)**			
No disability ×			
Less than recommended level (ref)			
Having disability ×	**1.84 (1.70, 1.97),** **<0.001**	**0.82 (0.65, 0.98),** **<0.001**	**0.013 (0.011, 0.015),** **<0.001**
Recommended level physical activity			

Abbreviations: ref, reference category; PCS, Physical Component Summary; MCS, Mental Component Summary; SF-6D, Short-Form Six-Dimension health index.

Models (1 to 3) were adjusted for age, gender, relationship status, highest education level completed, household yearly disposable income, labour force status, Indigenous status, region of residence, smoking status, and alcohol consumption.

Values in bold are statistically significant.

The association between disability and physical activity with the SF-36 domain scores is shown in Table 5. People with disabilities have considerably lower scores in each of the SF-36’s eight sub-scales: PF (b = -11.08), RP (b = -23.17), RE (b = -12.71), SF (b = -11.33), MH (b = -4.72), VT (b = -7.50), BP (b = -12.56), and GH (b = -9.61), according to the findings. However, undertaking recommended level of physical activity per week was linked to substantially higher scores in all dimensions of the SF-36: PF (b = 2.34), RP (b = 3.33), RE (b = 3.09), SF (b = 3.05), MH (b = 2.29), VT (b = 4.25), BP (b = 2.00), and GH (b = 4.05). Moreover, it is observed that PF (b = 4.83), RP (b = 7.25), RE (b = 4.78), SF (b = 4.15), MH (b = 1.43), VT (b = 1.81), BP (b = 2.67), and GH (b = 1.93) have increased in disabled adults who perform the recommended level of physical activity than their non-disabled counterparts who engaged in less than the recommended level of physical activity.

**Table 5:** The relationship between disability and physical activity with the dimensions of the SF-36, random-effects GLS regression.

Variables	Model 1: PF	Model 2: RP	Model 3: RE	Model 4: SF	Model 5: MH	Model 6: VT	Model 7: BP	Model 8: GH
	β (95% CI),	β (95% CI),	β (95% CI),	β (95% CI),	β (95% CI),	β (95% CI),	β (95% CI),	β (95% CI),
	P-value	P-value	P-value	P-value	P-value	P-value	P-value	P-value
**Disability**								
No (ref)								
Yes	**-11.08 (-11.29,**	**-23.17 (-23.53,**	**-12.71 (-13.08,**	**-11.33 (-11.57,**	**-4.72 (-4.89,**	**-7.50 (-7.69,**	**-12.56 (-12.79,**	**-9.61 (-9.77,**
	**-10.88), <0.001**	**-22.80), <0.001**	**-12.35), <0.001**	**-11.10), <0.001**	**-4.56), <0.001**	**-7.32), <0.001**	**-12.34), <0.001**	**-9.44), <0.001**
**Physical activity**								
Less than recommended level (ref)								
Recommended level	**2.34 (2.17, 2.50), <0.001**	**3.33 (3.03, 3.63), <0.001**	**3.09 (2.78, 3.39), <0.001**	**3.05 (2.85, 3.25), <0.001**	**2.29 (2.16, 2.43), <0.001**	**4.25 (4.10, 4.41), <0.001**	**2.00 (1.81, 2.19), <0.001**	**4.05 (3.91, 4.19), <0.001**
Interaction terms (Disability × physical activity)								
No disability × Less than recommended level (ref)								
Having disability × Recommended level physical activity	**4.83 (4.52, 5.14), <0.001**	**7.25 (6.68, 7.82), <0.001**	**4.78 (4.20, 5.35), <0.001**	**4.15 (3.78, 4.52), <0.001**	**1.43 (1.17, 1.68), <0.001**	**1.81 (1.52, 2.09), <0.001**	**2.67 (2.31, 3.02), <0.001**	**1.93 (1.67, 2.18), <0.001**

Abbreviations: ref, reference category; PF, Physical Functioning; RP, Role Physical; RE, Role Emotional; SF, Social Functioning; MH, Mental Health; V, Vitality; BP, Bodily Pain; GH, General Health

Models (1 to 8) were adjusted for age, gender, relationship status, highest education level completed, household yearly disposable income, labour force status, Indigenous status, region of residence, smoking status, and alcohol consumption.

Values in bold are statistically significant.

## Discussion

This study examined the association between disability and physical activity with HRQoL among the adult population in Australia using data from the HILDA survey. The findings showed that 27% of the adult population in Australia has some form of disability, and about 34% perform the recommended level of physical activity. The study findings revealed that disability and physical activity were independently associated with HRQoL. Specifically, people who had a disability and those who did not meet the recommended level of physical activity had substantially lower HRQoL than those who had no disability and those who completed the recommended level of physical activity. The nature of these relationships did not change even after controlling for potential confounding factors.

This study reveals a strong association between disability and a low score of HRQoL. The scores of HRQoL for the disabled on PCS, MCS, SF-6D and in each of the eight subscales of the SF-36 are smaller than those without disability. Similar associations between disability and HRQoL were reported in Australia [43] and North America [44]. One plausible reason for the association between disability and low HRQoL is the high perception of poor physical health due to the limitations in the performance of physical functions [43]. For example, an earlier study found that limitations in the performance of activities of daily living such as hygiene and dressing were major contributors to low HRQoL among persons with upper limb post-stroke spasticity [44]. Similarly, another study reported that limitations in self-care activities and functional mobility were the strongest predictors of poor HRQoL among people living with idiopathic Parkinson’s disease [45]. Since HRQoL largely represents people’s expectations of their health relative to what they actually experience, any perceived or actual limitation in bodily function will be seen as poor health and result in low HRQoL [46]. Thus, promoting active coping mechanisms with increased performance of physical activities, including activities of daily living, could improve physical functioning and HRQoL among persons with disabilities [47].

Also, findings from this study revealed that physical activity was positively associated with HRQoL. It means recommended levels of physical activity were associated with a significant increase in HRQoL and vice versa. Similar associations between physical activity and HRQoL were recorded in studies conducted in Spain [26], the UK and elsewhere [12, 48]. The probable reason could be regular physical activity enhances the sense of control, physical fitness and functioning [24, 49, 50]. Additionally, physical activity is associated with considerable mental stimulation and improvement in psychological health [49]. Perhaps, respondents who met the recommended level of physical activity in the current study might have gained some physical and psychological benefits associated with physical activity as manifested in the higher PCS, MCS, SF-6D and SF-36 scores compared to those who did not meet the recommended level of physical activity. Although this finding does not infer causality, it provides yet another strong evidence on the possible impact of physical activity on HRQoL.

### Strength and limitations

The main strength of this study is the large sample size and longitudinal nature of the study. This study utilizes 247,457 person-year observations using the most recent 19 waves of the HILDA survey spanning the period (2002–2020) to examine the association between disability and physical activity with HRQoL among adults in Australia. Despite these strengths, there are some limitations of this study. First, because the current study used unbalanced longitudinal datasets for the analysis, the findings only suggest an association between disability and physical activity with HRQoL, and inferences on causal relationships cannot be made based on the findings. However, the consistency of the current findings across different measures of HRQoL supports the need for further studies into the causal relationships between disability and physical activity with HRQoL using balanced longitudinal data. Second, previous studies reported that different forms of disability have a different impact on HRQoL [44]. However, in the present study, disability was not classified into forms or types, and therefore, the influence of different types of disability on physical activity or HRQoL could not be ascertained. Also, because the level of physical activity was self-reported, there is a possibility of social desirability bias which could affect the interpretation of the study findings. Additionally, one significant confounding variable in the association between physical activity and HRQoL is chronic disease [12]. However, this was not controlled for in the present study, limiting the interpretation of the study findings.

### Policy and public health implications

This study builds on existing knowledge about the relationship between disability and physical activity with HRQoL. The findings highlight the importance of physical activity as a public health strategy to improve the quality of life among persons with disabilities. Therefore, stakeholders need to design and implement intervention programs that will increase participation in physical activities among adults. Such intervention programs should be targeted at raising awareness of the potential benefit of physical activity to adults. Additionally, certain types of physical activities may not be appropriate for certain types of disabilities, limiting participation or endangering health. Hence, engaging health professionals in determining the appropriate type and amount of physical activity could enhance participation and ensure safety [21].

## Conclusion

This study provides further evidence on the association between disability and physical activity with HRQoL using 19 waves longitudinal data from the HILDA survey. The findings revealed that disability negatively contributed to HRQoL, but increasing physical activity levels were associated with significant improvement in HRQoL. Therefore, there is a need to enhance public health intervention programs such as educational campaigns and engaging health professionals to promote physical activity. This could increase participation and thereby improve HRQoL. However, to what extent a different pattern of physical activity involvement is associated with better HRQoL and what pattern of physiotherapy will be more fruitful is beyond the scope of this paper. This aspect can be a further research option to be explored by the scholars.

## Supporting information

S1 Appendix (DOCX)

## Abbreviation

BMI: Body Mass Index
HILDA: Household, Income and Labour Dynamics in Australia Survey
HRQoL: Health-related Quality of Life
PCS: Physical Component Summary
MCS: Mental Component Summary
SF-6D: Short-Form Six-Dimension
SF-36: 36-Item Short-Form Health Survey
