# Supplementary material for: Disability, physical activity, and health-related quality of life in Australian adults: An investigation using 19 waves of a longitudinal cohort
Source: PLoS One. 2022 May 12;17(5):e0268304. doi: 10.1371/journal.pone.0268304 (PMC9098066; doi:10.1371/journal.pone.0268304)

**Appendix A**

Figure 1: Mean SF-36 domain scores by disability status, waves 2002-2020


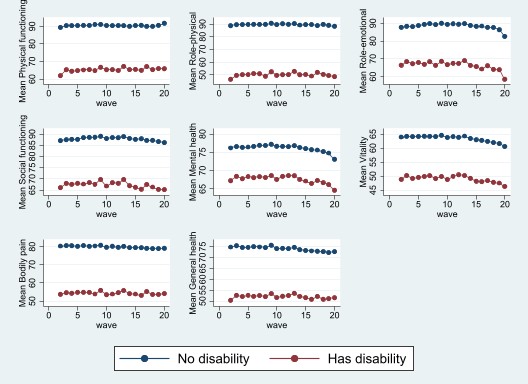


Figure 2: Mean SF-36 domain scores by physical activity, waves 2002-2020


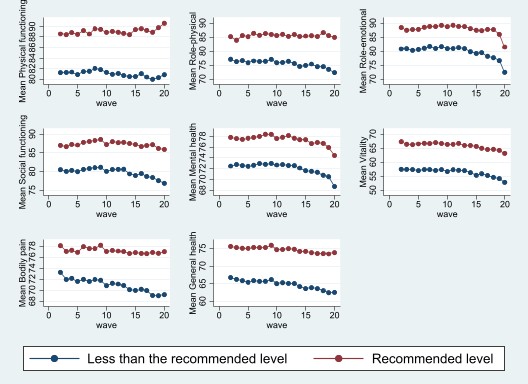

Supplement: S1 Appendix — (DOCX) [file pone.0268304.s001.docx]
